# Association of baseline hematoma and edema volumes with one-year outcome and long-term survival after spontaneous intracerebral hemorrhage: A community-based inception cohort study

**DOI:** 10.1177/1747493020974282

**Published:** 2020-11-25

**Authors:** James JM Loan, Angus B Gane, Laura Middleton, Brendan Sargent, Tom James Moullaali, Mark A Rodrigues, Laura Cunningham, Joanna Wardlaw, Rustam Al-Shahi Salman, Neshika Samarasekera, Western General Hospital, Edinburgh, Royal Infirmary of Edinburgh, St John’s Hospital, West Lothian, Astley Ainslie and Liberton Hospitals, Edinburgh

**Affiliations:** 1 Centre for Clinical Brain Sciences, The University of Edinburgh, Edinburgh, UK; 2 Department of Clinical Neurosciences, NHS Lothian, Edinburgh, UK; 3 UK Dementia Research Institute at Edinburgh, University of Edinburgh, UK; 4 Department of Neuroradiology NHS Lothian, Edinburgh, UK; 5 Edinburgh Medical School, The University of Edinburgh, Edinburgh, UK; *LATCH Collaborators are listed in the acknowledgments.

**Keywords:** Cohort study, intracerebral hemorrhage, outcome, peri-hematomal edema, radiology, survival

## Abstract

**Background:**

Hospital-based studies have reported variable associations between outcome after spontaneous intracerebral hemorrhage and peri-hematomal edema volume.

**Aims:**

In a community-based study, we aimed to investigate the existence, strength, direction, and independence of associations between intracerebral hemorrhage and peri-hematomal edema volumes on diagnostic brain CT and one-year functional outcome and long-term survival.

**Methods:**

We identified all adults, resident in Lothian, diagnosed with first-ever, symptomatic spontaneous intracerebral hemorrhage between June 2010 and May 2013 in a community-based, prospective inception cohort study. We defined regions of interest manually and used a semi-automated approach to measure intracerebral hemorrhage volume, peri-hematomal edema volume, and the sum of these measurements (total lesion volume) on first diagnostic brain CT performed at ≤3 days after symptom onset. The primary outcome was death or dependence (scores 3–6 on the modified Rankin Scale) at one-year after intracerebral hemorrhage.

**Results:**

Two hundred ninety-two (85%) of 342 patients (median age 77.5 y, IQR 68–83, 186 (54%) female, median time from onset to CT 6.5 h (IQR 2.9–21.7)) were dead or dependent one year after intracerebral hemorrhage. Peri-hematomal edema and intracerebral hemorrhage volumes were colinear (*R*^2^ = 0.77). In models using both intracerebral hemorrhage and peri-hematomal edema, 10 mL increments in intracerebral hemorrhage (adjusted odds ratio (aOR) 1.72 (95% CI 1.08–2.87); *p* = 0.029) but not peri-hematomal edema volume (aOR 0.92 (0.63–1.45); *p* = 0.69) were independently associated with one-year death or dependence. 10 mL increments in total lesion volume were independently associated with one-year death or dependence (aOR 1.24 (1.11–1.42); *p* = 0.0004).

**Conclusion:**

Total volume of intracerebral hemorrhage and peri-hematomal edema, and intracerebral hemorrhage volume alone on diagnostic brain CT, undertaken at three days or sooner, are independently associated with death or dependence one-year after intracerebral hemorrhage, but peri-hematomal edema volume is not.

**Data access statement:**

Anonymized summary data may be requested from the corresponding author.

## Background

Eighty-six percent of patients are dead or dependent within one year of a spontaneous (non-traumatic) intracerebral hemorrhage (ICH).^1^ Established predictors of poor outcome after ICH include greater baseline ICH volume, older age at onset, infratentorial location, and intraventricular extension.^2,3^ Peri-hematomal edema (PHE) is a radiological abnormality observed after ICH which occupies a variable volume.^4,5^

Within the first week of ICH onset, PHE manifests on CT brain scans as peri-hematomal hypoattenuation. However, the association between PHE on diagnostic brain CT and outcome is unclear. Of 16 cohort studies examining this association, all were hospital-based, 5 studies recruited participants prospectively,^6–10^ and only 4 studies adjusted for potential confounders.^7,11–13^ Eight studies stated the interval between symptom onset and brain imaging, which varied from 2^12^ to 24^14^ h.^6,7,10–12,14–16^ Eight studies found an association between PHE and poor outcome, of which none adequately adjusted for time from symptom onset to scan,^6–9,13,16–18^ three studies found that PHE was associated with better outcome,^11,19,20^ and five did not find an association.^10,12,14,15,21^

## Aims

We aimed to determine whether PHE measured on baseline (diagnostic) brain CT after ICH is associated with functional outcome and survival, independent of known predictors of outcome, in a community-based study.^3^

## Methods

### Design

We performed a prospective community-based inception cohort study to identify and follow adults aged ≥16 years who were resident in the Lothian Health Board region of Scotland at the time of ICH.

### Patients and follow-up

**Figure 1.**
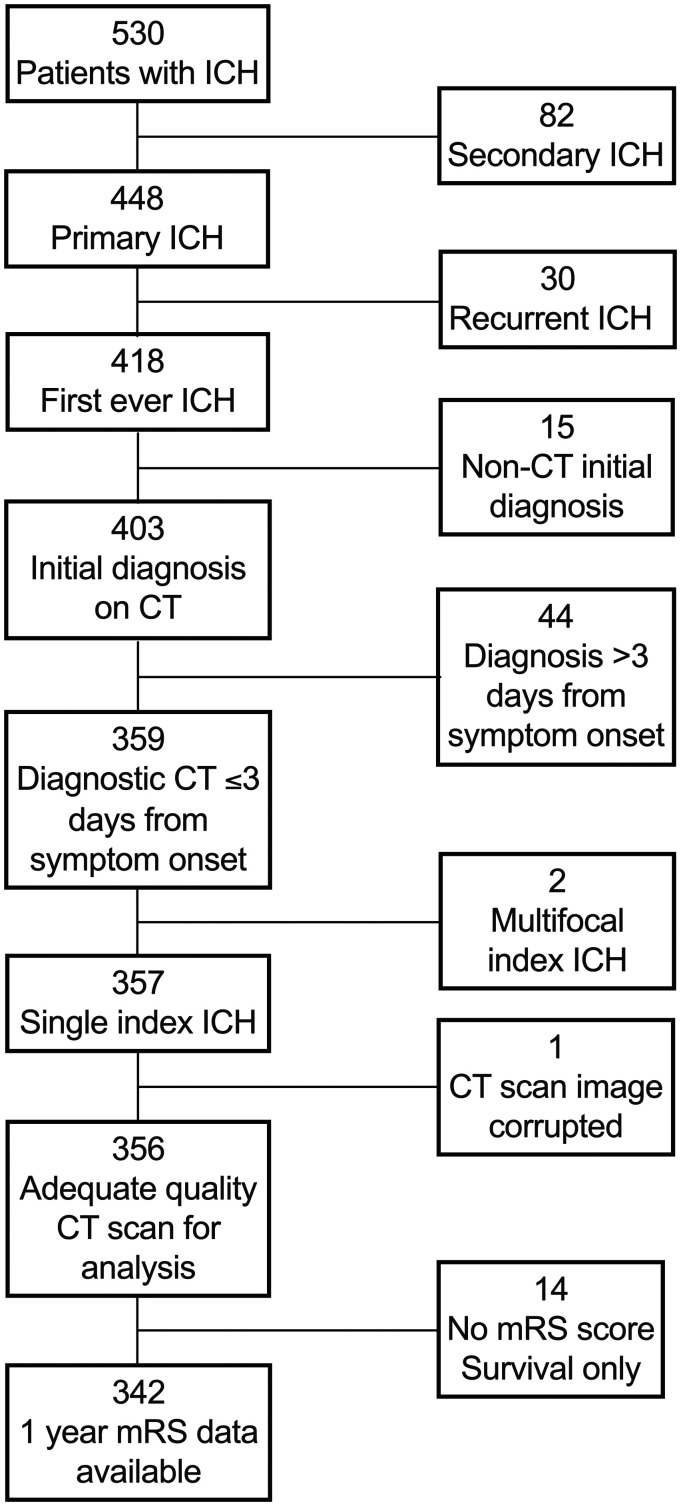
Patient selection flow diagram. ICH: intracerebral hemorrhage; CT: computed tomography brain scan; mRS: modified Rankin Scale.

The Lothian Audit of the Treatment of Cerebral Haemorrhage (LATCH) ascertained all patients with first-ever spontaneous ICH onset between 1 June 2010 and 31 May 2013 while resident in the Lothian Health Board region of Scotland, using hot pursuit of multiple overlapping sources, as described previously.^1^ Patients with secondary ICH (due to trauma, cerebral infarction or underlying macrovascular lesion) or who underwent diagnostic CT scan later than three days after symptom onset were excluded. Other exclusion criteria are detailed in Figure 1. We collected baseline demographic and clinical variables, including drugs which might affect ICH or PHE volume by interviewing patients or their families at the time of presentation and reviewing primary care and hospital records. We used multiple sources of follow-up including those used for case ascertainment and questionnaires sent annually to each participant's GP to determine their vital status and function according to the modified Rankin Scale (mRS) score at one year. Survival was established by surveillance of hospital and primary care.

### Imaging

A neuroradiologist (MAR) reformatted the first diagnostic non-contrast CT brain images into standard axial, coronal and sagittal planes of 5 mm slice thickness, MAR assessed ICH location according to The Cerebral Haemorrhage Anatomical RaTing inStrument (CHARTS)—from which lobar, deep, and infratentorial groups were derived—and the presence of intraventricular, subarachnoid or subdural extension masked to clinical characteristics and outcome.^22^ Each scan was assessed by two of four raters (JJML, ABG, LM, BS) who measured volumes of ICH and PHE on each scan independently, masked to clinical characteristics and outcome using Horos Viewer (v3.3.5, Nimble Co LLC 2018).^23^ They manually defined a region of interest (ROI) including ICH and PHE but excluding ventricles, skull, and previous stroke/small vessel disease on every slice showing ICH or PHE of every scan. In this fashion, intraventricular and extra-axial extension was excluded from analysis. Within the included ROI, raters separately segmented ICH and PHE in a semi-automated fashion by growing regions from manually selected seeds using thresholds of 40–100 Hounsfield Units (HU) for ICH and 5–33 HU for PHE.^23,24^ Individual raters adjusted thresholds from these initial values manually to correct outlines where the prespecified threshold incorrectly identified the PHE edge. To determine intra-rater agreement when measuring PHE, we first conducted a pilot of 40 scans with each rater analyzing 20 scans twice, with a two-week minimum interval between ratings. Inter-rater agreement was determined using the complete cohort. Because ICH and PHE volume are often correlated, PHE extension distance (EED) has been proposed as a measure of PHE which is less correlated with ICH volume.^5,25^ We measured EED in centimeters (cm) (Supplementary material).^5^ We calculated lesion volume as the sum of ICH and PHE volumes.

### Outcomes

General practitioners rated functional outcome using the mRS on annual postal questionnaires.^26^ The inception point was ICH symptom onset and follow-up continued until death or most recent completed annual follow-up questionnaire.

### Statistical analysis

We conducted analyses using Excel (v.16.37, Microsoft 2020), Prism (v8.4.2, Graphpad 2020), and R Core (v3.6.2, 2019) run on R Studio (v 1.1.464, Boston 2015). As there is no universally accepted method for determining sample size in multivariable regression, we used the maximum available sample size obtainable for the study period.^27^ We determined intra- and inter-rater agreement using mixed and random effects models, respectively, measured by intraclass correlation (ICC).^28^ We prespecified outcome measures and primary multivariable analyses in a written protocol (Supplementary material). For each case, we used the mean ICH volume and PHE volume measured by two raters for analyses. We dichotomized mRS for analysis into 0–2, signifying good outcome, and 3–6 signifying poor outcome (dead or dependent).

We conducted univariate comparisons between patients with or without the primary outcome at one year using Fisher's exact, Mann Whitney U, or unpaired *t* tests, as appropriate. We used log rank tests to test for univariate associations of quartiles of total lesion volume with long-term survival. We used simple linear and cubic regression to assess collinearity and determined *R*^2^ using the least-squares method. We calculated completeness of follow-up for functional outcome at one year by dividing the sum of follow-up times (days) by the sum of potential follow-up to death or the end of the one year follow-up period.^29^

We conducted multivariable analyses of associations with mRS at one year using binomial logistic regression with the primary independent variables of PHE volume, EED or ICH volume. As few patients were missing follow-up data at one year, these cases were dropped from this analysis and no data imputed. Two prespecified analyses included EED and ICH volume and PHE and ICH volume as primary independent variables. After finding PHE and ICH volumes were collinear, we performed exploratory analyses using total lesion volume as the primary independent variable. Since any association between PHE and functional outcome may vary by ICH location, we performed subgroup analyses of the lobar ICH subgroup and the subgroup with deep or infratentorial ICH combined.^30^ We also undertook a supplementary stratified analysis by restricting to quartiles of ICH volume. We adjusted all analyses for the following prespecified covariates which were derived from the ICH score: infratentorial ICH location, age at symptom onset, admission Glasgow Coma score (trichotomized 3–4; 5–12; 13–15); and the presence of intraventricular hemorrhage (IVH).^3^ As time from ICH symptom onset to diagnostic CT scan is associated with greater PHE volume within the first three days of onset, we undertook one further sensitivity analysis using the additional covariable of time (per 6 h, or fraction thereof) from symptom onset or when last seen well to CT.^31^ Cases with missing data were omitted. We calculated adjusted odds ratios (aORs) and hazard ratios (aHRs) per 10 mL increase in ICH, PHE and total lesion volume. Model fit was assessed using the Akaike information criterion (AIC), area under the receiver operating characteristic curve (AUC) measured by C-statistic, and the Hosmer–Lemeshow test.

We attempted survival analysis of the entire follow-up period available for each patient using Cox proportional hazards modeling, with the same independent variables as our logistic regression models. However, χ^2^ analysis of Schoenfeld residuals indicated non-proportional hazards (Supplementary figure). Using Kaplan–Meier analysis we determined that this was due to high hazards of death ≤30 days post-ICH. We therefore considered 30-day case fatality as a binary outcome using logistic regression and used a Cox regression model to analyze long-term survival in the cohort of patients who survived beyond 30 days after ICH.^32^ As just one patient with a baseline GCS 3–4 survived to 30 days, we dichotomized GCS (3–12 vs. 13–15) for these analyses. The distribution of Schoenfeld residuals in this model indicated that the assumption of proportionality was met. We used the AUC and Wald test to compare Cox models.

### Regulatory approval

LATCH was approved by the NHS Caldicott Guardian. Data were prospectively input to the audit database according to an a priori prepared protocol. For these analyses, we used anonymized data only, as such, no ethics committee approval was required. Similarly, no consent was required.

## Results

Of 530 patients with incident ICH (Figure 1), 418 had first-ever ICH without an identified underlying structural cause, 359 were diagnosed by brain CT on day three or earlier after symptom onset. After excluding two patients with multifocal ICH and one with a CT scan unsuitable for analysis, we included 356 patients. The median duration of follow-up from study opening until death or last follow-up was 71 days (interquartile range (IQR) 3–1682) for the entire cohort, 2574 days (IQR 2141–2944) for patients scored mRS 0–2 at one year and 12 days (IQR 2–544) for patients scored mRS 3–6 at one year. Death and dependency at one year were assessed in 96% (342/356) of patients and survival data were 96% complete (805/835 years of potential follow-up from study opening to data extraction). Intra- and inter-rater agreement about PHE volume was excellent (ICC 0.93 and 0.98, respectively).

### Clinical characteristics and univariable analyses

The median age of this cohort was 78 years (IQR 68–83) and 193 (54%) were female (Table 1). The median time to CT scan from symptom onset or when last seen well was 6.5 h (IQR 2.9–21.7). Older age, pre-existing dementia, alcohol abstinence, antiplatelet use, and paracetamol use at ICH onset were associated with death or dependency at one year (Supplementary Table 1). In these univariate analyses, higher baseline ICH volume, PHE volume, EED, and intraventricular hemorrhage (IVH) on diagnostic brain CT were associated with one-year death or dependency (Table 1). Admission GCS score was also associated with one-year death or dependency (Table 1).

**Table 1.** Clinical characteristics of patients according to one-year functional outcome after first-ever spontaneous ICH.

Characteristic	All	Year 1 mRS unknown	Independence (mRS 0–2)	Death or dependence (mRS 3–6)	Unadjusted comparison mRS 0–2 vs. mRS 3–6
*n*	356	14	50	292	
Female sex	193 (54%)	7 (50%)	27 (54%)	159 (55%)	>0.999*
Median age (years)	78 (68–83)	78 (67–84)	69 (57–77)	79 (71–84)	<0.001†
Median time to CT scan (h)^a^	6.5 (2.9–21.7)	17 (7.5–24.9)	7.7 (3.1–27.4)	6.2 (2.8–18.9)	0.24†
ICH characteristics					
Median ICH volume (mL)	22 (8–51)	5 (4–13)	9 (3–14)	29 (9–61)	<0.001†
Median peri-hematomal edema volume (mL)	25 (12–48)	11 (8–20)	12 (5–18)	29 (14–55)	<0.001†
Median total lesion volume (mL)	48 (20–98)	16 (11–37)	23 (7–32)	58 (25–115)	<0.001†
Median ICH radius (cm)	1.8 (1.2–2.3)	1.1 (1.0–1.5)	1.3 (0.8–1.5)	1.9 (1.3–2.4)	<0.001†
Mean edema extension distance (cm)	3.1 (2.4–3.8)	2.3 (2.0–2.9)	2.5 (1.8–2.7)	3.3 (2.6–4.0)	<0.001‡
ICH location					0.43*
Lobar	170 (48%)	6 (43%)	22 (44%)	142 (49%)	
Deep supratentorial	138 (39%)	7 (50%)	23 (46%)	108 (37%)	
Infratentorial	48 (14%)	1 (7%)	5 (10%)	42 (14%)	
Initial Glasgow coma scale score					<0.001*
13–15	201 (57%)	13 (93%)	45 (90%)	143 (49%)	
5–12	117 (33%)	1 (7%)	4 (8%)	112 (38%)	
3–4	38 (11%)	0 (0%)	1 (2%)	37 (13%)	
Intraventricular hemorrhage	179 (50%)	1 (7%)	10 (20%)	168 (58%)	<0.001*
Subarachnoid hemorrhage	156 (44%)	4(29%)	16 (32%)	136 (47%)	0.065
Subdural hemorrhage	39 (11%)	1 (7%)	3 (6%)	35 (12%)	0.33
Deaths					
30-day case fatality	162 (46%)	0 (0%)	0 (0%)	162 (55%)	<0.001*
Deaths at end of study period	293 (82%)	11 (79%)	17 (34%)	265 (91%)	<0.001*

Data are presented as *n* (%), median (interquartile range), or mean (standard deviation).

^a^Data missing for four cases (2 cases mRS 0–2; 2 cases mRS ≥ 3).

*χ^2^/Fisher's exact; †Mann Whitney; ‡unpaired *t* test.

### Collinearity of ICH and PHE metrics

**Figure 2.**
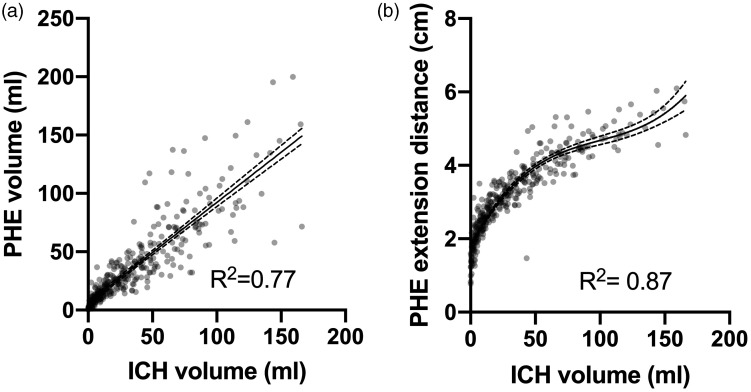
Scatterplots and regression analyses of ICH volume vs. PHE volume (a) or EED (b). Dots represent individual patients, solid lines represent the modeled regression line, and dashed lines indicate 95% confidence intervals. *R*^2^ represents the proportion of the variance for the dependent variable that is explained by the independent variable in the regression models.

PHE and ICH volumes (*R*^2^ = 0.77)) were highly correlated (Figure 2(a)). ICH volume and EED also highly correlated according to a cubic function (*R*^2^ = 0.87; Figure 2(b)).

### Functional outcome

We prespecified two multivariable models of functional outcome at one year. Our first prespecified model (Table 2) demonstrated no significant independent association between PHE volume and death or dependency but a significant association between ICH volume and death or dependency was identified after adjustment. Our second model also demonstrated no significant association between EED or ICH volume and death or dependency (Table 2).

**Table 2.** Initial prespecified logistic regression models of death or dependency at one year after first-ever spontaneous ICH

Independent variables	aOR	95% Confidence interval	*p* value	Area under ROC curve	Akaike information criterion
Peri-hematomal edema volume					
Intercept	0.017	0.00058–0.63	0.015	0.87	213.3
PHE volume (per 10 mL)	0.92	0.63–1.45	0.69		
ICH volume (per 10 mL)	1.72	1.083–2.87	0.029		
Age (per year)	1.072	1.04–1.11	<0.001		
IVH	3.44	1.49–8.64	0.005		
Infratentorial	1.89	0.64–6.56	0.27		
GCS 3–4					
GCS 5–12	1.29	0.047–14.39	0.85		
GCS 13–15	0.45	0.019–4.062	0.54		
Edema extension distance					
Intercept	0.010	0.00025–0.48	0.013	0.87	213.0
EED (per cm)	1.38	0.92–2.24	0.17		
ICH volume (per 10 mL)	1.38	0.56–3.39	0.48		
Age (per year)	1.070	1.040–1.10	<0.001		
IVH	3.49	1.51–8.75	0.005		
Infratentorial	2.16	0.71–7.76	0.20		
GCS 3–4					
GCS 5–12	1.20	0.44–13.4	0.90		
GCS 13–15	0.41	0.017–3.70	0.49		

Adjusted odds ratio (OR) indicates adjusted risk of poor outcome (mRS 3–6) associated with increasing ICH or PHE volume (per 10 mL), OED (per cm), age (per year), in the presence of IVH or infratentorial ICH, or GCS group (vs. GCS 3–4).

Both models exhibited instability, which sensitivity analyses showed was attributable to collinearity between ICH and PHE metrics. These sensitivity analyses with just one ICH or PHE metric per regression model showed significant associations between one-year death or dependency and greater ICH volume (aOR 1.58 (1.26–2.09); *p* < 0.001), PHE volume (aOR 1.47 (1.19–1.89); *p* = 0.001) and EED (aOR 2.52 (1.63–4.06); *p* < 0.001; Supplementary Table 2).

Because PHE volume and EED were collinear with ICH volume, we summed PHE and ICH volumes to derive total lesion volume, a measure of total parenchymal abnormality. Ten-milliliter increments in total lesion volume, yearly increases in age, and the presence of intraventricular hemorrhage were associated with one-year death and dependency, independent of other known predictors of outcome (Table 3).

**Table 3.** Final logistic regression model of death and dependency at one-year after first-ever spontaneous ICH

Independent variables	aOR	95% Confidence interval	*p* value	Area under ROC curve	Akaike information criterion
Intercept	0.0056	0.00015–0.74	<0.001	0.86	213.2
Total lesion volume (per 10 mL)	1.24	1.11–1.42	<0.001		
Age (per year)	1.069	1.039–1.10	<0.001		
IVH	3.63	1.58–9.04	0.004		
Infratentorial	2.055	0.71–7.70	0.21		
GCS 3–4					
GCS 5–12	1.17	0.045–12.28	0.90		
GCS 13–15	0.39	0.017–3.23	0.45		

Adjusted OR indicates adjusted risk of poor outcome (mRS 3–6) associated with increasing total lesion volume (per 10 ml), age (per year), in the presence of IVH or infratentorial ICH, or GCS group (versus GCS 3–4).

We conducted an additional exploratory subgroup analysis of functional outcome at one year in 30-day survivors. In this subgroup, outcome was not independently associated with lesion volume (aOR 1.007 (0.998–1.30); *p* = 0.073; Supplementary Table 3). Subgroup analyses stratified by ICH volume and lobar versus deep/infratentorial ICH demonstrated similar characteristics except that in small ICH volumes, the associations of EED (aOR 9.036 (1.086–110.22); *p* = 0.059) and PHE volume (aOR 13.7 (1.20–268.51); *p* = 0.058) approached, but did not achieve, statistical significance (Supplementary Tables 4 to 9). In a model using total lesion volume and the same covariables as previously described, we undertook a further exploratory sensitivity analysis using time (in hours) from ICH symptom onset to diagnostic CT scan as a supplementary covariable. This demonstrated similar characteristics with a significant association between longer time to CT and lower odds of one-year death or dependency (aOR 0.87 (0.78–0.97; per 6-h increment); *p* = 0.011; Supplementary Table 10).

### Survival

**Figure 3.**
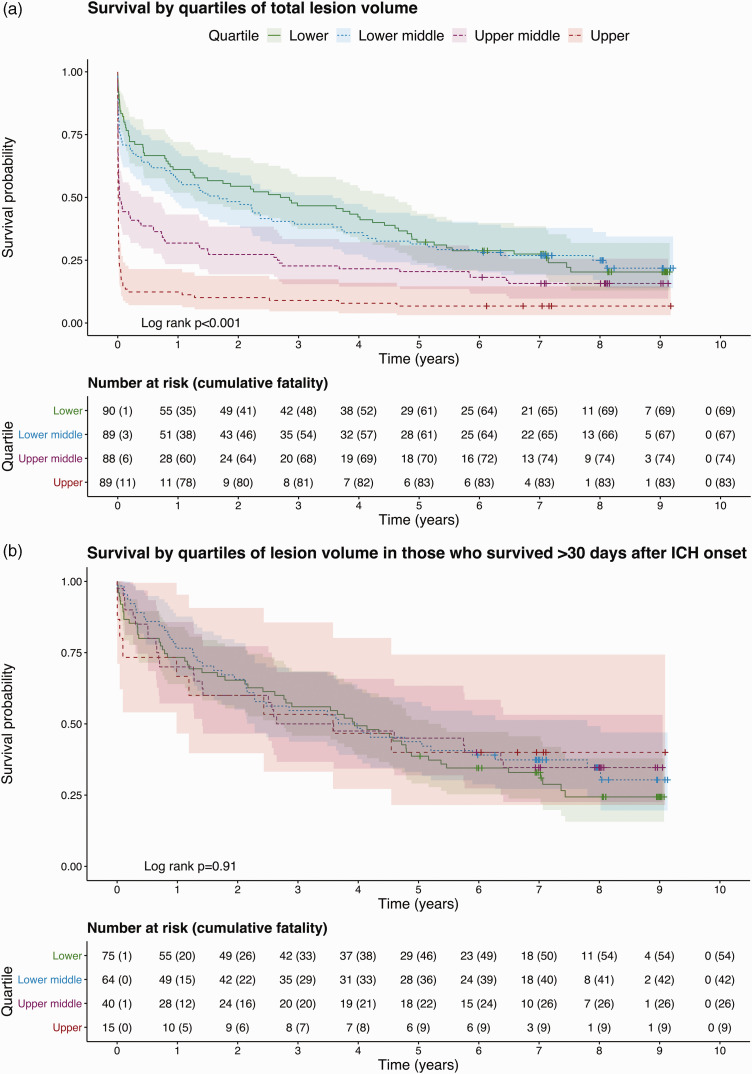
Kaplan–Meier curves of survival over up to nine years of follow-up in quartiles of lesion volume in the entire cohort (a) or restricted to 30-day survivors (b). Shaded areas indicate 95% confidence intervals.

One hundred sixty-two (46%) patients died within 30 days of ICH symptom onset and 293 (82%) died during the entire follow-up period (Table 1; Figure 3). Total lesion volume was independently associated with 30-day case fatality (Table 4; Figure 3). In the cohort of patients who survived >30 days from their ICH, lesion volume was not independently associated with long-term survival (Table 4; Figure 3).

**Table 4.** Multivariable analysis of deaths after first-ever spontaneous ICH

Independent variables	aOR/aHR	95% Confidence interval	*p* value	Area under ROC curve	Akaike information criterion/Wald
30-day case fatality: logistic regression					
Intercept	0.12	0.0069–3.75	0.16	0.89	309.6
Total lesion volume (per 10 mL)	1.22	1.15–1.30	<0.001		
Age (per year)	1.025	1.0012–1.051	<0.042		
IVH	2.82	1.57–5.10	<0.001		
Infratentorial	3.44	1.48–8.10	0.004		
GCS 3–4					
GCS 5–12	0.09	0.0044–0.57	0.035		
GCS 13–15	0.025	0.0012–0.15	0.001		
Death in 30-day survivors: Cox proportional hazards					
Total lesion volume (per 10 mL)	1.010	0.95–1.063	0.80	0.67	*p* = 2 × 10^−9^
Age (per year)	1.063	1.044–1.081	<0.001		
IVH	1.40	0.96–2.055	0.088		
Infratentorial	1.17	0.70–1.99	0.55		
GCS 3–12					
GCS 13–15	0.94	0.58–1.52	0.80		

Analysis of deaths up to 30 days after ICH by logistic regression: aOR indicates adjusted risk of death by 30 days; Akaike information criterion analysis of fit. Analysis of time to death in 30-day survivors by Cox proportional hazards; adjusted hazard ratio (HR) indicates adjusted hazards of death associated with increasing total lesion volume (per 10 mL), age (per year), in the presence of IVH or infratentorial ICH, or GCS group (versus GCS 3–4). Wald *p* value.

## Discussion

In this prospective, community-based cohort study of patients scanned in a median time of 6 h after ICH symptom onset, we did not find evidence of an association between any measure of baseline PHE volume with functional outcome that was independent of ICH volume, because ICH volume and measures of PHE were collinear. Total lesion (ICH and PHE) volume was associated with poor functional outcome at one year and 30-day case fatality after ICH. We found that 30-day survivors had similar probabilities of having a good outcome at one year and long-term survival irrespective of lesion volume on diagnostic CT brain scan.

### Comparison with other studies

Of four previous studies which have examined the association between PHE at baseline and poor outcome after ICH using multivariable analyses to adjust for ICH volume and other confounders, two found an association (133^7^ and 106^13^ patients), one did not find an association (596 patients)^12^ and one other (98 patients) found that greater relative PHE—the ratio of PHE:ICH volume—was associated with better survival at 90 days.^11^ We assessed outcome at one year and long-term survival whereas previous studies considered outcome at 14,^7^ 30,^13^ or 90 days.^11,12^ The previous reports of an association between PHE and poor outcome may be due to handling of ICH volume as a dichotomous variable,^7^ or measurement of maximal PHE area on a single axial image.^13^ Alternatively, selection bias may contribute to our differing findings; since both studies which found an association had hospital-based cohorts with smaller ICH volumes, for which the effect of PHE may be more pronounced.^7,13,33^ We did not observe a statistically significant independent effect of PHE volume on outcome in our analysis stratified by ICH volume, although the effect of PHE approached significance in the subgroup with smaller ICH volumes. The use of relative PHE may produce disproportionately high PHE measurements in smaller ICH volumes and consequent association with good outcome.^5,11^

### Strengths and limitations

We used a prospective community-based design with multiple sources of case ascertainment and prospective follow-up to identify adults with first ever spontaneous ICH at or very shortly after ICH onset, thus minimizing selection bias. Interventions which could affect PHE were infrequently utilized in our cohort and were spread similarly between outcome groups. Further, our population was not drawn from a trial and so there is low risk of interventions distorting outcomes. Our sample size was sufficient to permit our pre-specified multivariable analyses. Our images were reformatted into standardized axial, coronal, and sagittal plains for analysis, which was undertaken in a standardized fashion blind to clinical outcome. Imaging features of ICH were classified by a neuroradiologist and we were able to demonstrate high inter-observer agreement for the assessment of PHE, indicating precision with minimal measurement error. We prespecified the covariables used for adjustment in our multivariable analyses using known independent predictors of 30-day outcome after ICH, described by the ICH score which have been robustly established and validated.^3,34^ Other variables, such as systolic blood pressure,^21,35^ and blood glucose at initial medical assessment, as well as antithrombotic medication usage^36,37^ and diabetes mellitus,^38^ have been associated with various outcomes after ICH. However, these associations vary between reports or have not been shown to predict outcome independent of the ICH score components and were therefore not included in our multivariable analysis. By adjusting only for established predictors of outcome, while avoiding exploratory analyses, the risk of type two error or confounding obscuring our primary finding is minimized.

We assessed PHE volume at baseline, on the first CT scan done up to and including day three after ICH symptom onset. We did not perform serial imaging, which allows measurement of change in PHE volume over time. Rate of change in PHE volume could be associated with functional outcome independent of ICH growth rate and our present study was unable to determine this.^4,30^ We used CT as this is the most commonly used imaging modality after ICH, is better tolerated than MRI by elderly patients with acute ICH and is reliable for the semi-automated assessment of PHE.^24^ However, differentiating peri-hematomal hypodensity due to PHE from white matter lucencies related to small vessel disease is challenging on CT images.^24^ Because some of our predictor variables are widely used clinically predict outcome after ICH it is possible that they may have influenced clinicians decision making, thus becoming self-fulfilling predictors.^3^

## Conclusions

ICH volume and PHE measured on baseline CT scan are colinear. As such, we found that there was no association between baseline PHE volume and death or dependency at one year after ICH. Total lesion volume incorporates both ICH and PHE volume and was associated with death and dependency at 30 days and one year after ICH. Patients who survived the first month after ICH had similar risks of longer-term death or disability, irrespective of their initial total lesion volume. Future observational studies should consider serial imaging to determine whether differences in the evolution of ICH or PHE volumes over time are associated with long-term outcome.

## Supplemental Material

sj-zip-1-wso-10.1177_1747493020974282 - Supplemental material for Association of baseline hematoma and edema volumes with one-year outcome and long-term survival after spontaneous intracerebral hemorrhage: A community-based inception cohort study Click here for additional data file. Supplemental material, sj-zip-1-wso-10.1177_1747493020974282 for Association of baseline hematoma and edema volumes with one-year outcome and long-term survival after spontaneous intracerebral hemorrhage: A community-based inception cohort study by James JM Loan, Angus B Gane, Laura Middleton, Brendan Sargent, Tom James Moullaali, Mark A Rodrigues, Laura Cunningham, Joanna Wardlaw, Rustam Al-Shahi Salman and Neshika Samarasekera in International Journal of Stroke

## LATCH Collaborators

Western General Hospital, Edinburgh: Anne Addison, Kate Ahmad, Syed Alhadad, Peter Andrews, Elaine Bisset, Peter Bodkin, Ralph Bouhaidar, Paul Brennan, Brian Campbell, Siddharthan Chandran, Helen Cook, Richard Davenport, Martin Dennis, Chris Derry, Katrina Dodds, Fergus Doubal, Susan Duncan, Andrew Elder, Mike Fitzpatrick, Peter Foley, Ioannis Fouyas, Sudipto Ghosh, Rod Gibson, Claire Gordon, Robin Grant, Russell Hewett, Fiona Hughes, Mark Hughes, David Hunt, Neil Hunter, James Ironside, Imran Liaquat, Colin Josephson, Anant Kamat, Susan Kealley, Sarah Keir, Gillian Kerr, Simon Kerrigan, Peter Keston, Matthew King, Richard Knight, Elizabeth Macdonald, Graham Mackay, Donald Macleod, Malcolm Macleod, Conor Maguire, Steven Makin, Ashok Mathews, Fiona Maxwell, Stuart McClellan, Tracey Millar, Zoe Morris, Tim Morse, Colin Mumford, Katherine Murray, Lynn Myles, Graham Nimmo, Yi Ng, Suvankar Pal, Kristiina Rannikmae, Jonathan Rhodes, Jerard Ross, Tim Russell, Peter Sandercock, Robin Sellar, Mano Shanmuganathan, Himanshu Shekhar, Henry Simms, Mara Sittampalam, Colin Smith, Hamza Soleiman, Helen Spiers, Patrick Statham, Neo Stavrinos, Jon Stone, Joyce Stuart, Cathie Sudlow, David Summers, Pat Taylor, Antonia Torgersen, Margarethe van Dijke, Robert Walker, Belinda Weller, William Whiteley, Ian Whittle, Robert Will, Wendy Young.

Royal Infirmary of Edinburgh: Judith Anderson, Seona Broadbent, Laura Butler, Dave Caesar, Patricia Cantley, Jonathan Carter, Gareth Clegg, Andrew Coull, Alastair Crosswaite, James Dear, Simon Dummer, Fiona Duncan, Trish Elder-Gracie, Kate Enright, Tom Fitzgerald, Jane Fothergill, Brian Frier, David Grant, Alasdair Gray, Simon Hart, Robin Henderson, Alan Jaap, Simon Leigh-Smith, Michael Jones, Moyra Masson, Lynn McCallum, Martin McKechnie, Graham McKillop, Gillian Mead, Wendy Morley, Billie Morrow, Frank Morrow, Jon Murchison, Ross Murphy, Jasmine Ng, Olayinka Ogundipe, Dilip Patel, Alison Pollock, Matthew Reed, Geraint Roberts, Johann Selvarajah, Randy Smith, Claire Stirling, Neil Turner, Matthew Wilson, Stanko Yordanov.

St John’s Hospital, West Lothian: Nicola Bell, Sarah Chambers, Sandra Dewar, Donald Farquhar, Ali Harmouche, Ashok Jacob, Katherine Jackson, Anne Knox, Jon McCafferty, Sam Moultrie, Latana Munang, Donald Noble, Scott Ramsay, Linda Spence, Bethany Threlfall, Andrew Williams, James Wilson.

Astley Ainslie and Liberton Hospitals, Edinburgh: Alastair Fitzgerald, Andrew Jamieson, Peter Lange, Andrew McIntosh, Lewis Morrison, Iain Todd.
