# Supplementary material for: Association of baseline hematoma and edema volumes with one-year outcome and long-term survival after spontaneous intracerebral hemorrhage: A community-based inception cohort study
Source: Int J Stroke. 2020 Nov 25;16(7):828–39. doi: 10.1177/1747493020974282 (PMC8521378; doi:10.1177/1747493020974282)
Supplement: sj-zip-1-wso-10.1177_1747493020974282 - Supplemental material for Association of baseline hematoma and edema volumes with one-year outcome and long-term survival after spontaneous intracerebral hemorrhage: A community-based inception cohort study [file sj-zip-1-wso-10.1177_1747493020974282.zip › Supplementary materials - tables_clean.docx]

|  | | | | | |
| --- | --- | --- | --- | --- | --- |
| **Characteristic** | **All** | **Year 1 mRS unknown** | **Slight/no dependence (mRS 0-2)** | **Death or dependence (mRS 3-6)** | **p: mRS 0-2 vs. mRS 3-6** |
| n | 356 | 14 | 50 | 292 |  |
| Ischaemic stroke^a^ | 58 (16%) | 0 (0%) | 4 (8%) | 54 (18.5%) | 0.069* |
| Transient ischaemic attack^a^ | 42 (12%) | 1 (7%) | 6 (12%) | 35 (12.0%) | >0.999* |
| Dementia and cognitive decline |  |  |  |  | **0.003*** |
| Not diagnosed | 44 (12%) | 0 (0%) | 1 (2%) | 43 (15%) |  |
| Dementia | 35 (10%) | 2 (14%) | 1 (2%) | 32 (11%) |  |
| Diagnosis of cognitive decline without dementia | 277 (78) | 12 (86%) | 48 (96%) | 217 (74%) |  |
| Diabetes mellitus^a^ |  |  |  |  | 0.200* |
| None | 314 (88%) | 12 (86%) | 48 (96%) | 254 (87%) |  |
| Type 1 | 1 (0%) | 0 (0%) | 0 (0%) | 1 (0%) |  |
| Type 2 | 40 (11%) | 2 (14%) | 2 (4%) | 36 (12%) |  |
| Hypertension^a^ | 237 (67%) | 11 (79%) | 27 (54%) | 199 (68%) | 0.053* |
| Smoking status^b^ |  |  |  |  | 0.437* |
| Never | 148 (45%) | 6 (43%) | 25 (50%) | 117 (40%) |  |
| Previous smoker | 128 (36%) | 4 (29%) | 16 (32%) | 108 (37%) |  |
| Current smoker | 78 (22%) | 4 (29%) | 9 (18%) | 65 (22%) |  |
| Alcohol intake |  |  |  |  |  |
| Drinks no alcohol (teetotal) | 182 (51%) | 7 (50%) | 14 (28%) | 161 (55%) | **0.0004*** |
| Median non-teetotal alcohol intake (units/week$)$ | 12 (24) | 18 (28) | 10 (17.3) | 12 (23) | 0.314† |
| Antihypertensive prescribed |  |  |  |  | 0.306* |
| None | 178 (54%) | 7 (50%) | 30 (60%) | 141 (48%) |  |
| One | 84 (24%) | 4 (29%) | 9 (18%) | 71 (24%) |  |
| More than one | 94 (26%) | 3 (21%) | 11 (22%) | 80 (27%) |  |
| Antiplatelet prescribed prior to onset |  |  |  |  | **0.042*** |
| None | 200 (61%) | 10 (71%) | 35 (70%) | 155 (53%) |  |
| One | 141 (40%) | 4 (29%) | 12 (24%) | 125 (43%) |  |
| More than one | 15 (4%) | 0 (0%) | 3 (6%) | 12 (4%) |  |
| Anticoagulant prescribed prior to onset | 46 (13%) | 3 (21%) | 5 (10%) | 38 (13%) | 0.651* |
| Statin prescribed prior to onset | 138 (39%) | 5 (36%) | 13 (26%) | 120 (41%) | 0.059* |
| Oral corticosteroid prescribed prior to onset | 11 (3%) | 0 (0%) | 1 (2%) | 10 (3%) | >0.999* |
| NSAID prescribed prior to onset | 129 (36%) | 2 (14%) | 15 (30%) | 112 (38%) | 0.273* |
| Non-NSAID, non-steroidal immunomodulator prescribed prior to onset | 8 (2%) | 0 (0%) | 1 (2%) | 7 (2%) | >0.999* |
| Paracetamol prescribed prior to onset | 83 (23%) | 2 (14%) | 2 (4%) | 79 (27%) | **0.0001*** |
| Neurosurgery (evacuation or ventricular drain) | 16 (5%) | 3 (21%) | 2 (4%) | 11 (4%) | >0.999* |
| Osmotic therapy prescribed after onset | 3 (1%) | 0 (0%) | 1 (2%) | 2 (1%) | 0.38 |

**Supplementary table 1: Medical history and medications at ICH onset.** Data are presented as n(%), median (interquartile range), or mean (standard deviation). *$\chi$^2^/Fisher’s exact, † Mann Whitney, ‡ unpaired t-test, a data missing for one case (mRS$\boldsymbol{\geq}$3), b data missing for 2 cases (mRS$\boldsymbol{\geq}$3)

| **Independent variables** | **Odds ratio** | **Confidence interval** | **p-value** | **AUC** | **AIC** |
| --- | --- | --- | --- | --- | --- |
| ***Model 3*** | | | | | |
| *Intercept* | 0.017 | 0.00061-0.64 | 0.016 | 0.87 | 211.5 |
| ICH volume | 1.58 | 1.26-2.085 | 0.00031 |  |  |
| Age | 1.071 | 1.040-1.10 | <0.0001 |  |  |
| IVH | 3.47 | 1.50-8.69 | 0.0051 |  |  |
| Infratentorial | 1.95 | 0.67-6.70 | 0.25 |  |  |
| GCS 3-4 |  | | |  |  |
| GCS 5-12 | 1.26 | 0.047-13.88 | 0.87 |  |  |
| GCS 13-15 | 0.44 | 0.018-3.88 | 0.52 |  |  |
| ***Model 4*** | | | | | |
| *Intercept* | 0.0052 | 0.00015-0.22 | 0.0031 | 0.87 | 213.3 |
| OED | 2.52 | 1.63-4.056 | <0.0001 |  |  |
| Age | 1.068 | 1.038-1.10 | <0.0001 |  |  |
| IVH | 3.66 | 1.59-9.14 | 0.0034 |  |  |
| Infratentorial | 2.45 | 0.80-8.88 | 0.14 |  |  |
| GCS 3-4 |  | | |  |  |
| GCS 5-12 | 0.99 | 0.039-9.97 | 0.99 |  |  |
| GCS 13-15 | 0.30 | 0.013-2.39 | 0.34 |  |  |
| ***Model 5*** | | | | | |
| *Intercept* | 0.029 | 0.0013-0.99 | 0.027 | 0.86 | 216.7 |
| PHO volume | 1.47 | 1.19-1.89 | 0.0012 |  |  |
| Age | 1.066 | 1.037-1.099 | <0.0001 |  |  |
| IVH | 3.90 | 1.71-9.62 | 0.0018 |  |  |
| Infratentorial | 2.054 | 0.70-7.070 | 0.21 |  |  |
| GCS 3-4 |  | | |  |  |
| GCS 5-12 | 1.045 | 0.043-9.90 | 0.97 |  |  |
| GCS 13-15 | 0.32 | 0.015-2.34 | 0.34 |  |  |

**Supplementary table 2: Diagnostic logistic regression models of overall 1-year outcome.** Odds ratio (OR) indicates adjusted risk of poor outcome (mRS$\boldsymbol{\geq}$3) associated with increasing ICH or PHO volume (per 10ml), OED (per cm), age (per year), in the presence of IVH or infratentorial ICH, or GCS group (versus GCS 3-4). AUC area under the receiver operating characteristic curve; AIC Akaike information criterion.

| **Independent variables** | **Odds ratio** | **Confidence interval** | **p-value** | **AUC** | **AIC** |
| --- | --- | --- | --- | --- | --- |
| *Intercept* | 0.0016 | 0.000065-0.28 | <0.0001 | 0.78 | 187.3 |
| Lesion volume | 1.007 | 0.998-1.30 | 0.073 |  |  |
| Age | 1.073 | 1.041-1.11 | <0.0001 |  |  |
| IVH | 3.80 | 1.55-10.34 | 0.0056 |  |  |
| Infratentorial | 1.42 | 0.44-5.30 | 0.58 |  |  |
| GCS 3-12 |  | | |  |  |
| GCS 13-15 | 2.26 | 0.78-7.66 | 0.15 |  |  |

**Supplementary table 3: Logistic regression model of 1-year outcome in survivors of the first 30 days after ICH.** Odds ratio (OR) indicates adjusted risk of poor outcome (mRS$\boldsymbol{\geq}$3) associated with increasing ICH or PHO volume (per 10ml), OED (per cm), age (per year), in the presence of IVH or infratentorial ICH, or GCS group (versus GCS 3-4). AUC area under the receiver operating characteristic curve; AIC Akaike information criterion.

**Stratified models**

Lobar

| **Independent variables** | **Odds ratio** | **Confidence interval** | **p-value** | **AUC** | **AIC** |
| --- | --- | --- | --- | --- | --- |
| ***Model 1*** | | | | | |
| *Intercept* | 0.070 | 0.00037-8.97 | 0.29 | 0.86 | 107.9 |
| ICH volume | 1.63 | 0.997-3.045 | 0.083 |  |  |
| OED | 0.89 | 0.27-2.86 | 0.85 |  |  |
| Age | 1.03 | 0.98-1.084 | 0.23 |  |  |
| IVH | 5.22 | 1.21-37.33 | 0.048 |  |  |
| Infratentorial | NA | NA | NA |  |  |
| GCS 3-4 |  | | |  |  |
| GCS 5-12 | 2.93 | 0.062-88.14 | 0.55 |  |  |
| GCS 13-15 | 1.94 | 0.050-53.98 | 0.70 |  |  |
| ***Model 2*** | | | | | |
| **Independent variables** | **Odds ratio** | **Confidence interval** | **p-value** | **AUC** | **AIC** |
| *Intercept* | 0.055 | 0.00031-5.45 | 0.23 | 0.86 | 107.9 |
| ICH volume | 1.69 | 1.021-3.056 | 0.61 |  |  |
| PHO volume | 0.93 | 0.62-1.51 | 0.74 |  |  |
| Age | 1.031 | 0.98-1.086 | 0.22 |  |  |
| IVH | 5.22 | 1.20-37.56 | 0.0495 |  |  |
| Infratentorial | NA | NA | NA |  |  |
| GCS 3-4 |  | | |  |  |
| GCS 5-12 | 2.94 | 0.063-83.53 | 0.54 |  |  |
| GCS 13-15 | 1.93 | 0.050-50.70 | 0.70 |  |  |

**Supplementary table 4: Stratified logistic regression models of overall 1-year outcome in patients with lobar ICH.** No cases of infratentorial ICH in this cohort. OR indicates adjusted risk of poor outcome associated with increasing ICH or PHO volume (per 10ml), OED (per cm), age (per year), in the presence of IVH or infratentorial ICH, or GCS group (versus GCS 3-4).

Deep/infratentorial

| **Independent variables** | **Odds ratio** | **Confidence interval** | **p-value** | **AUC** | **AIC** |
| --- | --- | --- | --- | --- | --- |
| ***Model 1*** | | | | | |
| *Intercept* | 0.00021 | 0.0000018-0.013 | 0.00018 | 0.91 | 103.9 |
| ICH volume | 1.43 | 0.68-4.058 | 0.43 |  |  |
| OED | 1.91 | 0.37-8.95 | 0.42 |  |  |
| Age | 1.11 | 1.064-1.17 | <0.0001 |  |  |
| IVH | 1.65 | 0.50-5.83 | 0.42 |  |  |
| Infratentorial | 1.43 | 0.35-6.64 | 0.63 |  |  |
| GCS 3-12 |  | | |  |  |
| GCS 13-15 | 13.067 | 1.95-275.16 | 0.027 |  |  |
| ***Model 2*** | | | | | |
| **Independent variables** | **Odds ratio** | **Confidence interval** | **p-value** | **AUC** | **AIC** |
| *Intercept* | 0.00063 | 0.000015-0.015 | <0.0001 | 0.91 | 104.3 |
| ICH volume | 2.71 | 0.86-12.17 | 0.15 |  |  |
| PHO volume | 0.74 | 0.22-2.30 | 0.62 |  |  |
| Age | 1.12 | 1.068-1.18 | <0.0001 |  |  |
| IVH | 1.49 | 0.45-5.28 | 0.52 |  |  |
| Infratentorial | 1.019 | 0.26-4.41 | 0.98 |  |  |
| GCS 3-12 |  | | |  |  |
| GCS 13-15 | 13.010 | 1.94-277.75 | 0.028 |  |  |

**Supplementary table 5: Stratified logistic regression models of overall 1-year outcome in patients with infratentorial or deep ICH**. OR indicates adjusted risk of poor outcome associated with increasing ICH or PHO volume (per 10ml), OED (per cm), age (per year), in the presence of IVH or infratentorial ICH, or GCS group (versus GCS 3-12).

Size – lower

| **Independent variables** | **Odds ratio** | **Confidence interval** | **p-value** | **AUC** | **AIC** |
| --- | --- | --- | --- | --- | --- |
| ***Model 1*** | | | | | |
| *Intercept* | 0.000080 | 0.000000041-0.027 | 0.0052 | 0.84 | 86.7 |
| ICH volume | 0.77 | 0.0064-90.71 | 0.92 |  |  |
| OED | 9.036 | 1.085-110.22 | 0.059 |  |  |
| Age | 1.075 | 1.024-1.13 | 0.0053 |  |  |
| IVH | 3.65 | 0.72-25.054 | 0.14 |  |  |
| Infratentorial | 4.95 | 1.060-31.49 | 0.059 |  |  |
| GCS 3-4 |  | | |  |  |
| GCS 5-12 | 5.62 | 0.055-742.74 | 0.48 |  |  |
| GCS 13-15 | 1.64 | 0.027-89.41 | 0.82 |  |  |
| ***Model 2*** | | | | | |
| **Independent variables** | **Odds ratio** | **Confidence interval** | **p-value** | **AUC** | **AIC** |
| *Intercept* | 0.0013 | 0.0000020 – 0.21 | 0.023 | 0.84 | 86.3 |
| ICH volume | 0.62 | 0.0045-77.75 | 0.85 |  |  |
| PHO volume | 13.67 | 1.20-268.51 | 0.058 |  |  |
| Age | 1.072 | 1.021-1.13 | 0.0078 |  |  |
| IVH | 3.52 | 0.71-23.86 | 0.15 |  |  |
| Infratentorial | 5.20 | 1.12-32.76 | 0.051 |  |  |
| GCS 3-4 |  | | |  |  |
| GCS 5-12 | 5.40 | 0.054-714.02 | 0.49 |  |  |
| GCS 13-15 | 1.43 | 0.023-77.037 | 0.87 |  |  |

**Supplementary table 6: Stratified logistic regression models of overall 1-year outcome in patients in the lower ICH volume quartile**. OR indicates adjusted risk of poor outcome associated with increasing ICH or PHO volume (per 10ml), OED (per cm), age (per year), in the presence of IVH or infratentorial ICH, or GCS group (versus GCS 3-4).

Size – lower middle

| **Independent variables** | **Odds ratio** | **Confidence interval** | **p-value** | **AUC** | **AIC** |
| --- | --- | --- | --- | --- | --- |
| ***Model 1*** | | | | | |
| *Intercept* | 0.0000037 | <0.0001-0.0013 | 0.0075 | 0.90 | 68.9 |
| ICH volume | 0.75 | 0.063-7.66 | 0.81 |  |  |
| OED | 6.17 | 0.37-164.62 | 0.23 |  |  |
| Age | 1.11 | 1.047-1.21 | 0.0028 |  |  |
| IVH | 8.97 | 1.64-81.65 | 0.025 |  |  |
| Infratentorial | 1.96 | 0.20-44.69 | 0.60 |  |  |
| GCS 3-12 |  | | |  |  |
| GCS 13-15 | 7.0x10^7^ | <0.0001-inf | 0.99 |  |  |
| ***Model 2*** | | | | | |
| **Independent variables** | **Odds ratio** | **Confidence interval** | **p-value** | **AUC** | **AIC** |
| *Intercept* | 0.00012 | 0.000000087-0.029 | 0.0048 | 0.90 | 68.8 |
| ICH volume | 0.85 | 0.087-7.51 | 0.88 |  |  |
| PHO volume | 2.082 | 0.69-8.098 | 0.23 |  |  |
| Age | 1.11 | 1.046-1.21 | 0.0029 |  |  |
| IVH | 8.76 | 1.62-77.62 | 0.025 |  |  |
| Infratentorial | 2.017 | 0.20-46.19 | 0.58 |  |  |
| GCS 3-12 |  | | |  |  |
| GCS 13-15 | 0.00000064 | <0.0001-inf | 0.99 |  |  |

**Supplementary table 7: Stratified logistic regression models of overall 1-year outcome in patients in the lower-middle ICH volume quartile**. OR indicates adjusted risk of poor outcome associated with increasing ICH or PHO volume (per 10ml), OED (per cm), age (per year), in the presence of IVH or infratentorial ICH, or GCS group (versus GCS 3-12).

Size – upper middle

| **Independent variables** | **Odds ratio** | **Confidence interval** | **p-value** | **AUC** | **AIC** |
| --- | --- | --- | --- | --- | --- |
| ***Model 1*** | | | | | |
| *Intercept* | 1764.64 | 0.47-2.36x10^7^ | 0.089 | 0.81 | 51.4 |
| ICH volume | 0.58 | 0.13-2.31 | 0.45 |  |  |
| OED | 0.23 | 0.022-2.18 | 0.18 |  |  |
| Age | 1.035 | 0.96-1.12 | 0.37 |  |  |
| IVH | 2.18 | 0.25-22.89 | 0.48 |  |  |
| Infratentorial | 0.39 | 0.022-10.48 | 0.51 |  |  |
| GCS 3-12 |  | | |  |  |
| GCS 13-15 | 0.73 | 0.090-5.56 | 0.76 |  |  |
| ***Model 2*** | | | | | |
| **Independent variables** | **Odds ratio** | **Confidence interval** | **p-value** | **AUC** | **AIC** |
| *Intercept* | 42.93 | 0.084-48539.26 | 0.26 | 0.78 | 52.1 |
| ICH volume | 0.47 | 0.11-1.67 | 0.26 |  |  |
| PHO volume | 0.80 | 0.52-1.27 | 0.29 |  |  |
| Age | 1.035 | 0.96-1.12 | 0.36 |  |  |
| IVH | 2.35 | 0.28-23.66 | 0.32 |  |  |
| Infratentorial | 0.46 | 0.030-11.77 | 0.58 |  |  |
| GCS 3-12 |  | | |  |  |
| GCS 13-15 | 0.72 | 0.090-5.37 | 0.74 |  |  |

**Supplementary table 8: Stratified logistic regression models of overall 1-year outcome in patients in the upper-middle ICH volume quartile**. OR indicates adjusted risk of poor outcome associated with increasing ICH or PHO volume (per 10ml), OED (per cm), age (per year), in the presence of IVH or infratentorial ICH, or GCS group (versus GCS 3-12).

Size – upper

| **Independent variables** | **Odds ratio** | **Confidence interval** | **p-value** | **AUC** | **AIC** |
| --- | --- | --- | --- | --- | --- |
| ***Model 1*** | | | | | |
| *Intercept* | NA | NA | NA | NA | NA |
| ICH volume | NA | NA | NA |  |  |
| OED | NA | NA | NA |  |  |
| Age | NA | NA | NA |  |  |
| IVH | NA | NA | NA |  |  |
| Infratentorial | NA | NA | NA |  |  |
| GCS 3-12 |  | | |  |  |
| GCS 13-15 | NA | NA | NA |  |  |
| ***Model 2*** | | | | | |
| **Independent variables** | **Odds ratio** | **Confidence interval** | **p-value** | **AUC** | **AIC** |
| *Intercept* | NA | NA | NA | NA | NA |
| ICH volume | NA | NA | NA |  |  |
| PHO volume | NA | NA | NA |  |  |
| Age | NA | NA | NA |  |  |
| IVH | NA | NA | NA |  |  |
| Infratentorial | NA | NA | NA |  |  |
| GCS 3-12 |  | | |  |  |
| GCS 13-15 | NA | NA | NA |  |  |

**Supplementary table 9: Stratified logistic regression models of overall 1-year outcome in patients in the upper ICH volume quartile**. Analysis was not possible as no patients in this cohort had a good outcome at one-year post-ICH. OR indicates adjusted risk of poor outcome associated with increasing ICH or PHO volume (per 10ml), OED (per cm), age (per year), in the presence of IVH or infratentorial ICH, or GCS group (versus GCS 3-12).

**Sensitivity analysis**

| **Independent variables** | **Odds ratio** | **Confidence interval** | **p-value** | **AUC** | **AIC** |
| --- | --- | --- | --- | --- | --- |
| *Intercept* | 0.0023 | 0.000046-0.12 | 0.0018 | 0.88 | 202.56 |
| Total lesion volume (per 10ml) | 1.28 | 1.13-1.48 | <0.001 |  |  |
| Time (per six hours) | 0.87 | 0.79-0.97 | 0.011 |  |  |
| Age | 1.08 | 1.05-1.12 | <0.001 |  |  |
| IVH | 3.63 | 1.54-9.36 | 0.0048 |  |  |
| Infratentorial | 2.03 | 0.67-7.25 | 0.24 |  |  |
| GCS 3-4 |  | | |  |  |
| GCS 5-12 | 1.36 | 0.047-16.52 | 0.83 |  |  |
| GCS 13-15 | 0.49 | 0.019-4.84 | 0.60 |  |  |

**Supplementary table 10: Sensitivity analysis using final logistic regression models of overall 1-year outcome using additional covariable of time from last seen well or symptom onset.** OR indicates adjusted risk of poor outcome associated with increasing total lesion volume (per 10ml), time from onset (per six hours, or fraction thereof), age (per year), in the presence of IVH or infratentorial ICH, or GCS group (versus GCS 3-4). It wasn’t possible to establish time of symptom onset or when last seen well to the hour for four patients. These were dropped from this analysis.
