# Supplementary material for: Association of baseline hematoma and edema volumes with one-year outcome and long-term survival after spontaneous intracerebral hemorrhage: A community-based inception cohort study
Source: Int J Stroke. 2020 Nov 25;16(7):828–39. doi: 10.1177/1747493020974282 (PMC8521378; doi:10.1177/1747493020974282)
Supplement: sj-zip-1-wso-10.1177_1747493020974282 - Supplemental material for Association of baseline hematoma and edema volumes with one-year outcome and long-term survival after spontaneous intracerebral hemorrhage: A community-based inception cohort study [file sj-zip-1-wso-10.1177_1747493020974282.zip › Supplementary materials - protocol.docx]

# Background

1. Perihaematomal oedema (PHO) is visible on computed tomography (CT) and magnetic resonance imaging (MRI) in the majority of patients after intracerebral haemorrhage (ICH).
2. Perihaematomal hypodensity seen on CT brain is likely to reflect cytotoxic/ionic oedema in the first few hours after an ICH, followed by vasogenic oedema.^1^
3. PHO may contribute to poor outcome after ICH but the findings of studies examining the association between PHO and outcome differ. In a systematic review, we identified only six studies which were prospective and used a prespecified scanning protocol and there was heterogeneity in methods of measuring PHO.
4. PHO might be a potential therapeutic target after ICH

# Aims

1. Validate semiautomated quantification of PHO volume and oedema extension distance (OED) after ICH.
2. To determine if there is an association between:
   1. PHO/OED and death, after adjusting for other known predictors of outcome.
   2. PHO/OED and functional outcome at one year, after adjusting for other known predictors of outcome.

# Methods

## Study design

- 1. Prospective, population-based inception cohort of adults with spontaneous ICH in the Lothian health board region of Scotland
  2. Lothian Audit of the Treatment of Cerebral Haemorrhage (LATCH)

## Inclusion criteria

1. First-ever spontaneous ICH confirmed by CT head imaging or pathology (biopsy or post-mortem examination) or sudden deaths where a diagnosis of ICH was suspected but the diagnosis remained unconfirmed. We will exclude patients diagnosed without CT head.
2. Aged 16 years or over at the time of diagnosis.
3. Resident in the area served by the National Health Service (NHS) Lothian Health board at the time of the ICH.
4. Date of diagnosis 1st June 2010-31st May 2013.
5. No evidence of underlying cause (e.g. tumour, intracranial vascular malformation, venous thrombosis, prior trauma or haemorrhagic conversion of a cerebral infarct) other than cerebral small vessel diseases
6. Time to scan is 3 days or fewer from ICH symptomatic onset.

## Imaging characteristics

## ICH volume: ICH volume is defined as an area of hyperdensity representing acute blood products in the brain parenchyma on the diagnostic CT scan and will be calculated by semi-automated volumetric methods using Horos (MacOS): see [appendix](#_Appendix:_PHO_measurement) for a detailed description. Any regions of hypodensity found within high density haematoma will be considered to be ICH.

## PHO volume: PHO is defined as the hypodense area immediately adjacent to the ICH, but which does not represent cerebral small vessel disease, encephalomalacia, sulci, or the ventricular system. See [appendix](#_Appendix_1:_PHO) description of the methods for calculating PHO using manual and semi-automated methods in Horos. PHO measurements of interest are:

- Absolute PHO volume (mL): total lesion volume –ICH volume
- Oedema extension distance (OED), ICH radius and total lesion volume::

$$ICH radius={ICH}_{r}= \sqrt[3]{\frac{{ICH}_{v}}{\frac{4\pi}{3}}}$$

$$Lesion volume= {ICH}_{v}+{PHO}_{v}$$

$$Oedema extension distance=\sqrt[3]{\frac{Lesion volume}{\frac{4\pi}{3}}}- {ICH}_{r}$$

Where, ICH_v_= Mean ICH volume, PHO_v_= mean PHO volume.

## PHO: validation of techniques

Pilot

1. PHO measured using semi-automated and manual methods using Horos software for the first 40 cases included in the LATCH1 cohort.
2. This will be repeated after a delay of at least 2 weeks.
3. Each scan will be reviewed by two assessors blind to the other’s assessment and to clinical outcome.
4. Interclass correlation (ICC) will be calculated to determine intra- and inter-rater agreement in the measurements of PHO (absolute volume, relative volume and OED: one-way random effects ICC will be used for inter-rater agreement and two-way mixed effects ICC used to determine intra-rater agreement for individual raters.{Koo, 2016 #1814} . For this we will use the ICC function of the R package IRR (Various Coefficients of Interrater Reliability and Agreement; <https://www.rdocumentation.org/packages/irr/versions/0.84.1/topics/icc>)
5. Mean volumes for PHO and OED will be derived and agreement between manual and semi-automated methods assessed (ICC)

Go:Not Go

1. Before proceeding to analysis of the whole LATCH1 cohort we will ensure that the following criteria are met
   1. Inter-rater agreement: ICC >0.80
   2. Intra-rater agreement: ICC >0.80
2. If these criteria are not met, steps to further refine analysis protocol will be considered
3. If met, we will proceed to full analysis

## PHO measurement: data collection

1. Following satisfactory completion of the pilot, we will proceed to collection of PHO volumes from scans of all patients meeting inclusion criteria.
2. This will be undertaken using:
   1. Semi-automated method only
   2. Two reviewers per scan
3. ICC will be calculated to determine inter-rater agreement
4. A decision will be taken concerning whether PHO or OED will be used in future statistical analysis by considering inter- and intra-rater reliability as well as correlation with ICH volume: OED will be selected if this is demonstrated to be similarly reliable to PHO volume and is less correlated with ICH volume.

## Patient characteristics

1. Age at onset
2. Sex
3. Date of index ICH symptom onset
4. Time from symptom onset to baseline scan
5. Pre-ICH history of diabetes
6. Pre-ICH history of ischaemic stroke
7. Pre-ICH history of hypertension
   - Defined as either:
     1. History of hypertension in medical records before ICH
     2. Using antihypertensive medication at time of ICH
8. Atrial fibrillation
9. Antiplatelet medication use at time of ICH
10. Antithrombotic medication use at time of ICH
11. Use of a statin at the time of ICH
12. Use of steroids or other medications which could alter inflammation at the time of ICH
13. Pre-ICH blood pressure readings
14. GCS on admission
15. Systolic and diastolic BP on admission
16. ICH location (using CHARTS definitions)
17. Intraventricular extension
18. Pre-ICH ischaemic stroke

## Imaging biomarkers

1. CT SVD score - 1 point for each of the following if present:
   - Severe lucencies (Van Swieten Scale = 2) in anterior or posterior periventricular white matter
   - Lacunes  ≥2
   - Severe (=2) central or cortical atrophy

The combined 4-point ordinal score therefore assesses the global burden of SVD from 0 (no imaging features of severe SVD) to 3 (imaging features of SVD scored as severe for each imaging variable)

## Outcome measures

1. Modified Rankin Scale Score one year after ICH
   1. Assessed by the patient’s GP on completion of the patient’s annual postal questionnaire.
2. Death
   1. Date of death will be established using multiple sources as previously described:^2^ These include
      1. Patient’s hospital electronic patient record
      2. Patient’s GP
      3. General Register Office for Scotland
      4. The Office of the Procurator Fiscal

## Statistical analysis

### *Survival analysis*

If the assumption of proportional odds is met, the hazard of death before censoring at last follow-up (2010-present) will be modelled using Cox-regression analysis with the following covariables:

- - 1. PHO [measure to be specified] (see above)
    2. Age
    3. ICH volume on diagnostic CT head
    4. Intraventricular extension on diagnostic CT head (yes/no)
    5. ICH location
    6. GCS

Prior to model development the distribution of measures for each candidate coverable will be considered in exploratory univariate analyses to inform use of categorial vs. continuous data. Multivariable models will be fit to these data and sensitivity analyses used as appropriate to identify covariable-dependent effects of PHO on outcome.

### *Functional outcome*

Functional outcome following ICH at one year will be modelled using ordinal logistic regression. If likelihood ratio or Brandt testing indicate that the assumption of proportional odds for each outcome class has been violated then a binomial logistic regression model of odds of death or dependency (mRS>2) at one year will be used. For each model the following covariables will be used:

1. OED/PHO (see above)
2. Age
3. ICH volume
4. Intraventricular extension
5. ICH location
6. GCS

Prior to model development the distribution of measures for each candidate coverable will be considered in exploratory univariate analyses to inform use of categorial vs. continuous data. Multivariable models will be fit to these data and sensitivity analyses used as appropriate to identify covariable-dependent effects of PHO on outcome.

# Appendix 1: PHO measurement methods

## Manual PHO measurement

1. Horos programme
2. Identify the image for analysis
   1. Album = database
   2. Patient name ‘Latch Study’
   3. Patient ID 000XXX_LATCH
   4. Select ‘LINCHPIN 5mm Ax Mar image series’ by double click, new window with large image should appear
3. Manipulate the image so the ICH and PHO are in clear view
   1. Use toolbar icons above ‘mouse button function’ to select zoom function and other tools
4. Draw an outer region tracing just beyond the pixels that you perceive to be PHO on each image with PHO present. Be careful when encephalomalacia and/or the ventricles are present that these are excluded from this region. You can also exclude the skull and other hyperdense tissue that you don’t want the software to recognise as ICH too.
5. Open menu “ROI” -> “ROI manager”
   1. Highlight the text saying “unnamed” on the new window.
   2. Press backspace
   3. Type “Exclusion” in place
   4. Close ROI manager window
6. Menu “ROI” -> “Set Pixel Values to...”
   1. Ensure that the following are selected:
      1. ROIs with same name as the selected ROI
      2. Outside ROIs
      3. To this new value: the default value is okay. Ensure this is a negative number
   2. Finish by hitting enter or selecting “OK” and the area of the head outside the inclusion region drawn should now be blacked out
7. Right click on the image you are working on from the side bar list of sequences in order to pull up a side by side view one will be the unaltered full CT and the other the blacked out selection to work on edema segmentation. This will allow you to have a spatial view of the entire brain for reference when segmenting edema and choosing a threshold or area to segment.
8. Calculate ICH volume
   1. Click ‘ROI’ from dropdown menu at the top of the screen
   2. Select ‘Grow region (2D/3D segmentation)’ and configure the settings as shown, including naming the ROI ‘ICH’
   3. The settings on this page will automatically compute the volumes on each slice when the cursor is left-clicked on the ICH. If there are satellite areas that haven’t been covered, these can be picked up with an additional click. Where a) not all ICH, or conversely, b) surrounding brain is incorporated by mistake, delete the ROI on that series and adjust your threshold (decrease and increase lower thresholds respectively)
   4. Move through the image series, ensuring you’ve covered slice where there is blood
   5. Once you have grown/drawn a ROI named ‘ICH’ on all consecutive slices where there is blood, go to ROI -> ROI manager and a volume should appear. If a volume is not computed check ICH ROI appears **once** on all **consecutive** slices of interest
9. Calculate the total lesion volume manually
   1. Use the pencil tool (keyboard shortcut = D) to manually draw around the region of PHO, including the ICH on each image. You can adjust your drawing by using the repulsor tool (keyboard shortcut = R)
   2. Name each measurement ‘lesion’ by opening ROI manager and changing the name of “Unnamed” to “lesion”. Once one ‘lesion’ ROI is drawn on all consecutive slices where oedema is present, the total ‘lesion’ volume is computed in ROI manager (below).
10. Enter the ICH and total lesion volumes into the data collection spreadsheet: to calculate PHO via the manual method, simply subtract the ICH volume from the ‘lesion’ volume
11. Now save your ROIs:
    1. ROI -> Save all ROIs in this series -> study name with your initials as below (PHO analysis folder in documents)
    2. Should you need to reload your ROIs: ROI -> Import ROI(s) -> find your save ROIs

## Semi-automated PHO measurement

1. Open Horos
2. Identify the image for analysis
3. Manipulate the image so the ICH and PHO are in clear view
4. Use toolbar icons above ‘mouse button function’ to select zoom function and other tools
5. Draw an outer region tracing just beyond the pixels that you perceive to be PHO on each image with PHO present. Be careful when encephalomalacia and/or the ventricles are present that these are excluded from this region
6. Menu “ROI” -> “Set Pixel Values to...”
   1. Ensure that the following are selected:
      1. ROIs with same name as the selected ROI
      2. Outside ROIs
      3. To this new value: the default value is okay. Ensure this is a negative number
   2. Finish by hitting enter or selecting “OK” and the area of the head outside the inclusion region drawn should now be blacked out
7. Right click on the image you are working on from the side bar list of sequences in order to pull up a side by side view one will be the unaltered full CT and the other the blacked out selection to work on edema segmentation. This will allow you to have a spatial view of the entire brain for reference when segmenting edema and choosing a threshold or area to segment.
8. Calculate ICH volume
   1. Follow steps in [‘Manual PHO measurement’](#_Manual_PHO_measurement) and see image there for configuring your semi-automated ICH volume measurement
9. Calculate oedema volume
   1. Working on only the selected regions, go back up to the top menu again to “ROI” and then from the drop down select “Grow Region (2D/3D/ segmentation)…” Configure your box so that it looks as pictured
   2. Every time you right click over PHO, the ROI will automatically generate and merge with every subsequent click. If you need to up the threshold you can do so and the new ROI will overtake the old one, you cannot however replace a segmentation with a smaller threshold range. To do this you will need to right click over the PHO ROI and delete the whole ROI series in ROI manager and redo with the newer lower threshold.
   3. Continue until all slices are segmented.

References

1. Urday S, Kimberly WT, Beslow LA, et al. Targeting secondary injury in intracerebral haemorrhage--perihaematomal oedema. *Nat Rev Neurol.* 2015;11(2):111-122.

2. Samarasekera N, Fonville A, Lerpiniere C, et al. Influence of intracerebral hemorrhage location on incidence, characteristics, and outcome: population-based study. *Stroke.* 2015;46(2):361-368.
